# Functional Analysis and Tissue-Specific Expression of Calcitonin and CGRP with RAMP-Modulated Receptors CTR and CLR in Chickens

**DOI:** 10.3390/ani14071058

**Published:** 2024-03-30

**Authors:** Tianjiao Huang, Jiancheng Su, Xinglong Wang, Ningkun Shi, Xiao Zhang, Jiliang He, Juan Li, Jiannan Zhang, Yajun Wang

**Affiliations:** Key Laboratory of Bio-Resources and Eco-Environment of Ministry of Education, Animal Disease Prevention and Food Safety Key Laboratory of Sichuan Province, College of Life Sciences, Sichuan University, Chengdu 610065, China; 2021222040035@stu.scu.edu.cn (T.H.);

**Keywords:** calcitonin signaling, RAMP interactions, receptor pharmacology, tissue-specific expression

## Abstract

**Simple Summary:**

Our study provides an insightful look into the molecular interaction mechanisms of calcitonin and calcitonin gene-related peptide in chickens. The study highlights distinct pathways activated by these receptors and demonstrates that the calcitonin receptor and calcitonin receptor-like receptors have unique sensitivities to their ligands, which can be influenced by receptor activity-modifying proteins. Furthermore, an extensive expression analysis across chicken tissue revealed diverse roles for calcitonin and calcitonin gene-related peptide in avian biology. Our findings provide a fresh perspective on the evolutionary variations in hormone signaling between chickens and mammals, broadening the scope of knowledge in vertebrate endocrine systems.

**Abstract:**

Calcitonin (CT) and calcitonin gene-related peptide (CGRP) are critical regulators of calcium balance and have extensive implications for vertebrate physiological processes. This study explores the CT and CGRP signaling systems in chickens through cloning and characterization of the chicken calcitonin receptor (CTR) and calcitonin receptor-like receptor (CLR), together with three receptor activity-modifying proteins (RAMPs). We illuminated the functional roles for chickens between the receptors examined alone and in RAMP-associated complexes using luciferase reporter assays. Chicken CTRs and CLRs stimulated the cAMP/PKA and MAPK/ERK signaling pathways, signifying their functional receptor status, with CT showing appreciable ligand activity at nanomolar concentrations across receptor combinations. Notably, it is revealed that chicken CLR can act as a functional receptor for CT without or with RAMPs. Furthermore, we uncovered a tissue-specific expression profile for *CT*, *CGRP*, *CTR*, *CLR*, and *RAMP*s in chickens, indicating the different physiological roles across various tissues. In conclusion, our data establish a clear molecular basis to reveal information on CT, CGRP, CTR, CLR, and RAMPs in chickens and contribute to understanding the conserved or divergent functions of this family in vertebrates.

## 1. Introduction

Calcitonin (CT) and calcitonin gene-related peptide (CGRP) are key members of the calcitonin peptide family [1,2]. CT is secreted from the parafollicular cells or C cells of the thyroid in mammals [1], whereas in non-mammalian vertebrates including birds, calcitonin-producing cells are abundantly located in the ultimobranchial gland [3]. Across species from amphibians to mammals, CT predominantly functions as a hormone that inhibits bone resorption, leading to a decrease in blood calcium levels [4]. CGRP is a neuropeptide discovered in humans through molecular biology methods, notable for its potent vasodilator activity [2,5]. CGRP also has wide-ranging physiological functions that include roles in metabolism, blood pressure regulation, inflammatory response, and auditory nerve development and function [6]. In mammals, both *CT* and *CGRP* originate from the calcitonin gene (*CALCA*) through alternative splicing, a process influenced by developmental stages and tissue-specific factors [7]. Despite low primary amino acid sequence similarity (about 20%), they share conserved secondary structures, including a six- or seven-membered ring formed by disulfide bonds and an α-helix configuration, culminating in a categorization within the calcitonin family [8,9,10,11,12].

In mammals, the functions of CT and CGRP are mediated through their binding to calcitonin receptors generated by the genes *CALCR* (which codes for the calcitonin receptor, CTR) and *CALCRL* (which codes for the calcitonin receptor-like receptor, CLR, previously known as CRLR), respectively [13]. Both receptors contain a large N-terminal extracellular domain (ECD), which plays a crucial role in hormone recognition by binding to the C-terminal portion of the peptides [14]. The intracellular regions are coupled with Gs/Gi and Gq proteins, activating diverse signaling pathways, including the cAMP/PKA pathway, MAPK/ERK pathway, and calcium mobilization [15,16,17].

In addition to ligand-receptor interactions, the complexity of CTR and CLR function was further increased through interaction with three receptor activity-modifying proteins (RAMP1, RAMP2, and RAMP3) by modulating receptor phenotypes, including receptor trafficking, ligand binding, and signal transduction. When expressed alone in mammals, CTR primarily binds to CT and shows slight selectivity for CGRP [18,19]. However, its affinity for CGRP significantly increases in the presence of RAMP1 or RAMP3 [20,21]. In contrast, CLR, alone biologically inactive, remains in the endoplasmic reticulum without association with RAMPs. The translocation of CLR to the plasma membrane is critically dependent on RAMPs [22]. When associated with RAMP1, the CLR + RAMP1 complex becomes a high-affinity receptor for CGRP, while dimerization of CLR and RAMP2 creates a receptor that is highly responsive to the related peptide adrenomedullin. The CLR and RAMP3 complex confers a second adrenomedullin receptor that has some selectivity for CGRP [17,22]. As CTR and CLR are widely expressed, it has been proposed that the tissue-specific expression of the RAMPs regulates the site-specific bioactivity of CT and CGRP [6,23].

Apart from mammals, calcitonin family genes have also been found in non-mammalian vertebrates. In chickens, genes encoding *CT*, *CGRP*, *CTR*, *CLR*, and *RAMPs* have been predicted or identified [24,25]. Chickens display higher circulating calcium levels compared to other species, despite a lack of calcitonin receptors in their osteoclasts [26]. This suggests that in chickens, CT’s biological role may not be associated with bone metabolism. CTR presence in avian reproductive tissues influences the eggshell calcification process, as demonstrated in guinea fowl and chickens [27,28,29]. Limited research on CGRP in chickens has revealed its presence in various neuronal regions and suggested implications in neurotrophic functions and appetite regulation [30,31,32].

Despite the vast understanding of these peptides in mammals, the functions of the calcitonin family within avian species, particularly chickens, remain less clear. To address this knowledge gap, we concentrate on utilizing luciferase receptor assays to elucidate the modulatory effects of RAMPs on receptor activity, alongside RNA-seq analysis for a detailed expression profile of calcitonin gene family members in chickens. The study aims to expand our understanding of CT and CGRP peptides in avian species, clarify the complex signaling pathways of chicken CTR and CLR, and provide a solid molecular foundation for deciphering the physiological functions of the calcitonin family in birds.

## 2. Materials and Methods

### 2.1. Chemicals, Primers, Peptides, and Antibodies

All chemicals were purchased from Sigma-Aldrich (St. Louis, MO, USA). Restriction enzymes were obtained from Takara Biotechnology Co., Ltd. (Dalian, China), and the transfection reagent PEI MAX (Polyethylenimine Linear, MW 40000) was procured from Yeasen Biotechnology Co., Ltd. (Shanghai, China). The eukaryotic expression vector pcDNA3.1 (+) was acquired from Invitrogen (Carlsbad, CA, USA). RNAzol was obtained from the Molecular Research Center (Cincinnati, OH, USA). All primers utilized in this study were synthesized by Youkang Biotech Company Limited (Chengdu, China) and are detailed in Supplemental Table S1. Chicken CT and CGRP were synthesized utilizing solid-phase Fmoc chemistry at GL Biochem (Shanghai, China). The synthesized peptides have a purity exceeding 95% (measured via HPLC) and were structurally validated using mass spectrometry. Antibodies targeting total CREB (48H2) rabbit mAb (1:1000, #9197) and phosphorylated CREB (Ser133) (87G3) rabbit mAb (1:1000, #9198), were acquired from Cell Signaling Technology (CST, Beverly, MA, USA).

### 2.2. Extraction of Total RNA and Reverse Transcription

For the generation of cDNA templates, we collected tissues from three laying hens (Lohmann layer, 1-year-old) sourced from local commercial companies in Chengdu. Tissue samples from the hens were collected during their post-laying period. The chickens were euthanized, and various tissues (e.g., lungs, hypothalamus) were collected, quickly frozen, and ground with liquid nitrogen, and the powder was taken to extract total RNA from the chicken tissues with RNAzol (Molecular Research Center, Cincinnati, OH, USA) and dissolved in DEPC-treated H_2_O. All animal experiments conducted for this study were approved by the Animal Ethics Committee of the College of Life Sciences, Sichuan University.

Reverse transcription (RT) was performed after detecting the RNA concentration by mixing 2 μg of total RNA and 0.5 μg of oligo-deoxythymidine in a total volume of 5 μL of DEPC-treated H_2_O, reacting at 70 °C for 10 min, and placing on ice for 2 min. The first strand buffer was then mixed with 0.5 μL of deoxynucleotide triphosphate (dNTP), 0.5 μL of Moroni mouse leukemia virus (MMLV) reverse transcriptase (Takara), 2 μL of 5 × buffer, and 2 μL of DEPC-treated H_2_O in a total volume of 10 μL. The reaction was carried out at 42 °C for 90 min. The resulting product was diluted in 100 μL of MilliQ-H_2_O for subsequent PCR amplification of the target gene.

### 2.3. Cloning the cDNAs of Chicken CTR, CLR, RAMP1, RAMP2, and RAMP3

We accessed the chicken genome database at http://www.ensembl.org/gallus_gallus (accessed on 3 December 2021) to retrieve the sequences of the *CTR*, *CLR*, *RAMP1*, *RAMP2*, and *RAMP3* genes. Their sequences in chicken are labeled *CTR* (cCTR, XM_040664599.2), *CLR* (cCLR, NM_001163650.2), *RAMP1* (cRAMP1, XM_040703787.1), *RAMP2* (cRAMP2, NM_001397898.1) and *RAMP3* (cRAMP3, NM_001144973.3), respectively. Subsequently, gene-specific primers were designed based on these sequences and the coding regions of these genes were amplified by PCR using Platinum SuperFi II DNA polymerase (Thermo Fisher Scientific, Waltham, MA, USA) using cDNA from chicken tissues (e.g., lungs, hypothalamus) with high expression levels of these genes as templates. Following amplification, the coding regions were cloned into the pcDNA3.1 (+) expression vector and subjected to sequencing (Youkang, Chengdu, China).

### 2.4. Sequence Alignment and Analysis

The amino acid sequences of CT, CGRP, CTR, and CLR from various species were retrieved from the GenBank database (https://www.ncbi.nlm.nih.gov/genbank/ (accessed on 21 December 2021)). The ClustalW program was utilized to align the inferred amino acid sequences of chicken CT, CGRP, CTR, and CLR with the corresponding sequences of human, pig, mouse, duck, and zebrafish. The online protein topology prediction tool TMpred (http://embnet.vital-it.ch/software/TMPRED_form.html (accessed on 23 December 2021)) was employed to predict the potential transmembrane domains of chicken CTR and CLR.

### 2.5. Functional Characterization of the Chicken Calcitonin Family Receptors (CTR and CLR)

Chinese hamster ovary (CHO) cells were cultured in Dulbecco’s modified Eagle’s medium (DMEM) supplemented with 10% (vol/vol) fetal bovine serum (HyClone, Logan, UT, USA), 100 U/mL penicillin G and 100 μg/mL streptomycin. The cells were maintained in a 90 mm culture dish (Nunc, Rochester, NY, USA) and incubated at 37 °C with 5% CO_2_. To investigate the signaling pathways associated with each receptor in CHO cells, three different reporter systems (pGL3-CRE-luciferase, pGL4-SRE-luciferase, and pGL3-NFAT-RE-luciferase) were utilized to monitor intracellular cAMP/PKA and MAPK/ERK signaling pathways and calcium mobilization of the activated receptors, respectively [33,34].

Prior to the transfection procedure, the cells were initially cultured in 6-well plates (Nunc, Rochester, NY, USA), maintaining a seeding density of approximately 300,000 cells per well. Upon achieving approximately 70% confluence, the cells underwent a co-transfection process. This procedure entailed the introduction of 700 ng of various luciferase reporter constructs (namely, pGL3-CRE-luciferase, pGL4-SRE-luciferase, or pGL3-NFAT-RE-luciferase) combined with 200 ng of a specific receptor plasmid (CTR or CLR) and 1000 ng of either RAMP1, RAMP2, RAMP3 expression plasmids, or alternatively, the empty pcDNA3.1 (+) vector. A volume of 5.7 μL of PEI MAX was used in 200 μL of buffer following the manufacturer’s protocol. After 24 h of incubation, the transfected CHO cells were subcultured in 96-well plates and passaged for an additional 24 h before peptide treatment. Then, the medium from the 96-well plates was removed, and the cells were treated with chicken CT/CGRP-containing medium (or untreated medium as a control) at concentrations ranging from 10^−12^ M to 10^−6^ M for 6 h. Subsequently, the cells were harvested for luciferase assays by lysing them in 1 × passive lysis buffer. To measure the luciferase activity of the cell lysate, 40 μL of luciferase assay reagent (Promega, Madison, WI, USA) was mixed with the cell lysate following the manufacturer’s instructions. The fluorescence activity of the luciferase in each treatment group was measured using a multimode microplate reader (TriStar LB941, Berthold Technologies, Bad Wildbad, Germany). Finally, the fluorescence activity of each treatment group was expressed as a relative increase ratio compared to the control group [35,36].

### 2.6. Western Blot

To assess whether the activation of chicken CTR and CLR, along with the presence of RAMPs, can augment the phosphorylation levels of cAMP response element-binding protein (CREB), 200 ng of CTR or CLR expression plasmids were combined with 1000 ng of chicken RAMPs or an empty pcDNA3.1 (+) vector in a 100 μL buffer. The resulting mixture was then transfected into CHO cells in a 12-well plate (Nunc, Rochester, NY, USA) using 3.6 μL of PEI MAX transfection reagent. After a 24 h incubation period, the cells were exposed to chicken CT (10 nM) or CGRP (10 nM) for 10 min. Subsequently, the culture medium was removed, and the cells were lysed according to the manufacturer’s instructions. Western blot analysis was performed to quantify the levels of CREB and phosphorylated CREB (p-CREB) [36,37]. The optical densities were analyzed and normalized using the software, ImageJ 1.52a (National Institutes of Health, Bethesda, MD, USA). The relative expression of p-CREB/CREB was determined by calculating the ratio of the optical density to that of the control without peptide treatment (0 min).

### 2.7. Tissue-Specific Expression Analysis of Chicken CTR, CLR, RAMP1, RAMP2, and RAMP3 Using RNA-Seq Data

The RNA sequencing (RNA-seq) data utilized in this study are archived in the CNGB Sequence Archive (CNSA) of the China National GeneBank DataBase (CNGBdb) under accession number CNP0003404 [38,39]. Utilizing this extensive RNA-seq dataset, an accessible chicken tissue gene expression atlas (TGEA) has been developed and previously made public via a preprint [40]. The TGEA, offering insights into gene expression profiles, is available through the Shiny Server (https://chickenatlas.avianscu.com/ (accessed on 18 January 2022)), which provides functionalities for gene searches, downloading of detailed figures, data access, and links to additional resources.

The dataset comprises RNA samples from various tissues collected from six mature chickens (Lohmann layer, 1-year-old), encompassing both sexes (three females, three males). Tissue samples from the female chickens were collected during their post-laying period. A wide range of tissues were collected, such as the cerebrum, midbrain, cerebellum, hindbrain, hypothalamus, spinal cord, retina, anterior pituitary, heart, liver, spleen, lung, kidney, skin, muscle, visceral fat, abdominal fat (abdominal subcutaneous fat), thymus gland, thyroid gland, pancreas, adrenal gland, tongue, proventriculus, gizzard, crop, duodenum, jejunum, ileum, cecum, rectum, testis, ovary, uterus, infundibulum, and magnum. Tissue samples for the reproductive organs—uterus, infundibulum, magnum, ovaries, and testes—were obtained from three female or three male chickens, respectively. Our study presents a detailed analysis of tissue-specific expression patterns for selected chicken transcripts, such as *CT*, *CGRP*, *RAMP*s, *CTR*, and *CLR*, quantified in terms of transcripts per million (TPM).

### 2.8. Data Analysis

The data were analyzed by Student’s *t*-test (between two groups) or by one-way ANOVA followed by Dunnett’s test in GraphPad Prism 8 (GraphPad Software, San Diego, CA, USA). All in vitro experiments were repeated at least three times to ensure the reliability of the results, and representative data are reported.

## 3. Results

### 3.1. Characterization of the Coding Regions of Chicken CT, CGRP, CTR, and CLR

**Figure 1:**
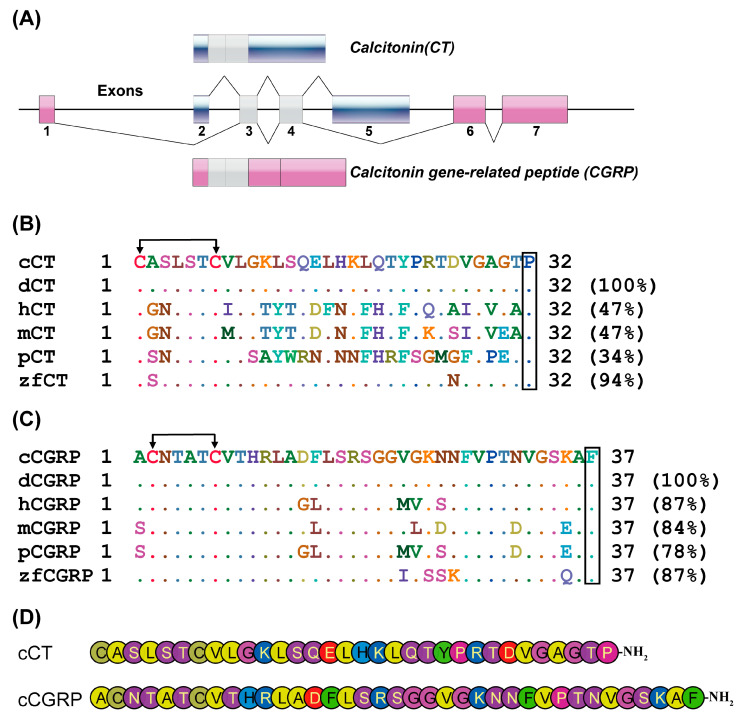
(**A**) Schematic diagram of alternative RNA processing of chicken *CALCA*. (**B**) Multiple sequence alignment of mature CT amino acids from six species. The NCBI accession numbers of these sequences are NP_001107180.1 (chicken), XP_027317826.1 (duck), NP_001029124.1 (human), NP_001292545.1 (mouse), NP_001098425.1 (pig), and XP_005172324.1 (zebrafish). (**C**) Multiple sequence alignment of mature CGRP amino acids from six species. The NCBI accession numbers of these sequences are NP_001258894.1 (chicken), XP_027317828.1 (duck), NP_000719.1 (human), NP_473425.2 (mouse), NP_001095943.1 (pig), and NP_001002471.1 (zebrafish). C: Conserved cysteines involved in disulfide bond formation, indicated by arrows. Conserved amidated residues at the C-terminus are indicated by squares, P: prolinamide site, F: phenylalanine amide site. (**D**) Amino acid sequences of chicken CT and CGRP (with amidated C-terminus) peptides used in this study.

Chicken *CT* and *CGRP* are generated by tissue-specific alternative splicing of chicken *CALCA* mRNA (Figure 1A). *CT* is formed by exons 2, 3, 4, and 5, while *CGRP* is generated by splicing exons 1, 3, 4, 6, and 7. As in mammals, chicken CT and CGRP consist of 32 and 37 amino acids, respectively. The analysis of the amino acid sequence alignment of chicken CT revealed a pronounced conservation of this molecule among non-mammalian vertebrates, while displaying significant divergence when compared to mammalian species. Specifically, the sequence identity of chicken CT with human, mouse, and pig was found to be 47%, 47%, and 34%, respectively (Figure 1B). This indicates a notable evolutionary divergence in the CT molecule between chickens and these mammals. In contrast, chicken CGRP exhibits a high degree of sequence similarity with mammalian species. Remarkably, the sequence identity of chicken CGRP with human, mouse, and pig is 87%, 84%, and 78%, respectively. This translates to a minor deviation of merely six to eight amino acids when compared with these mammalian species, underscoring a significant conservation of the CGRP molecule across different classes of vertebrates (Figure 1C). Both CT and CGRP peptides exhibit a cyclic structure consisting of six or seven members at their N-terminus, formed by disulfide bonds between cysteine residues, and possess an acylated C-terminus (Figure 1D). These structural features are crucial for the full activity of calcitonin family peptides and play a significant role in ligand-receptor binding.

**Figure 2:**
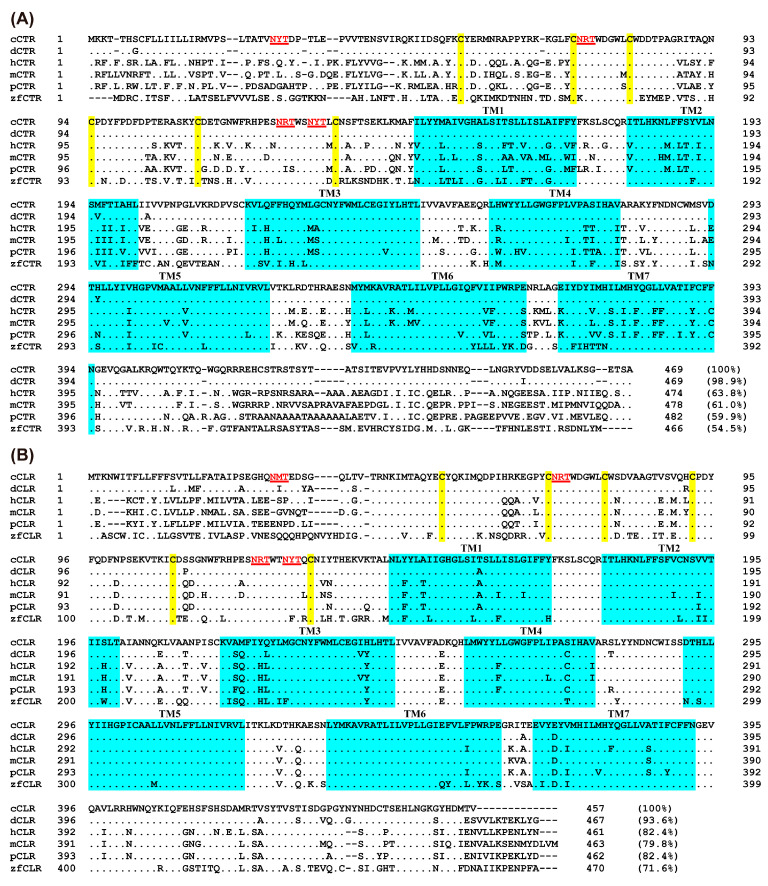
(**A**) Alignment of CTR amino acid sequences in six species. The NCBI accession numbers of these sequences are XP_040520533.1 (chicken), XP_027306590.2 (duck), NP_001158210.1 (human), AAK56134.1 (mouse), NP_999519.1 (pig), and XP_001920035.3 (zebrafish). (**B**) Alignment of CLR amino acid sequences in six species. The NCBI accession numbers of these sequences are NP_001157122.1 (chicken), XP_038037810.1 (duck), NP_001258680.1 (human), NP_061252.2 (mouse), Q8WN93.1 (pig), and NP_001004010.1 (zebrafish). TM1–7 (seven transmembrane domains) are shown in blue shading. The conserved cysteine residue C is shaded in yellow. Predicted N-glycosylation sites are indicated by red lines. “·” represents identical amino acids, and “-” denotes gaps in the sequence alignment.

Based on the predicted cDNA sequence or genomic sequence of chicken *CTR* and *CLR*, we cloned the cDNAs containing complete open reading frames (ORFs) of these genes from adult chicken hypothalamus or lung tissue by RT-PCR. The cloned chicken *CTR* and *CLR* cDNA coding regions are 1410 bp and 1374 bp in full length and predicted to encode proteins containing 469 and 457 amino acids, respectively. Figure 2A,B depict the results of amino acid sequence alignments of chicken CTR and CLR with their corresponding orthologues in various vertebrate species. The derived amino acid sequence from multiple sequence alignment of chicken CTR demonstrates a remarkably high homology with that of the duck, showing 99% similarity. When compared with other species, the homology percentages are 64% with human, 61% with mouse, 60% with pig, and 55% with zebrafish (Figure 2A). On the other hand, CLR in chickens exhibits a greater degree of sequence conservation across species compared to CTR. The sequence alignment for chicken CLR reveals significant homology with duck at 94%, and notably high similarities with human (82%), mouse (80%), pig (82%), and zebrafish (72%) (Figure 2B). This indicates that CLR, unlike CTR, maintains a more conserved amino acid sequence across a diverse range of vertebrate species.

The conservation of the seven transmembrane domains (TM1–TM7) is higher in these sequences compared to the N-terminal and C-terminal sequences. Specifically, the N-terminal and C-terminal sequences of the chicken CTR exhibit significant dissimilarities in comparison to non-avian species. Additionally, a closer examination of CTR and CLR sequences revealed several notable structural features. These features include a large N-terminal extracellular domain (ECD) that potentially functions as a binding pocket for peptides, containing six conserved cysteine residues that form three disulfide bonds to stabilize the N-terminal alpha-helical structure, four potential N-glycosylation sites (NXS/T, with X representing any amino acid except proline), seven conserved transmembrane domains, and a shorter C-terminal domain (Figure 2). These structural characteristics form the basis for receptor-ligand binding and signal transduction.

### 3.2. Overview of CTR/CLR Receptor and RAMP Interaction in Chickens

Despite the identification of two key receptors, CTR and CLR, along with three RAMPs within the calcitonin family in avian species, the specific functional roles of these receptors and their RAMP-associated complexes for CT and CGRP remain largely unexplored. To investigate the activation potential of synthetic chicken CT and CGRP on CTRs and CLRs in chickens and to examine the influence of various RAMPs on these receptor functions, CHO cells transiently expressing CTR/CLR-RAMP complexes were treated with these synthetic peptides. The induced signaling pathways following receptor activation were meticulously monitored using three cell-based luciferase reporter systems established in our laboratory: pGL3-CRE-luciferase, pGL4-SRE-luciferase, and pGL3-NFAT-RE-luciferase [41,42]. Table 1 displays the half-maximal effective concentration (EC_50_) values, reflecting the activation of CTR/CLR and RAMP complexes by chicken CT and CGRP in a dose-dependent manner. The findings delineate that our synthetically formulated chicken CT and CGRP are biologically active, as evidenced by their dose-dependent stimulation of luciferase activity in the CHO cells. Moreover, these peptides exhibit distinct activation profiles on the various receptor combinations expressed in the CHO cells, highlighting the nuanced interplay between the peptides and receptor complexes.

### 3.3. Effect of CT/CGRP on CTR/CLR–RAMP Complexes in cAMP/PKA Signaling Pathway

**Figure 3:**
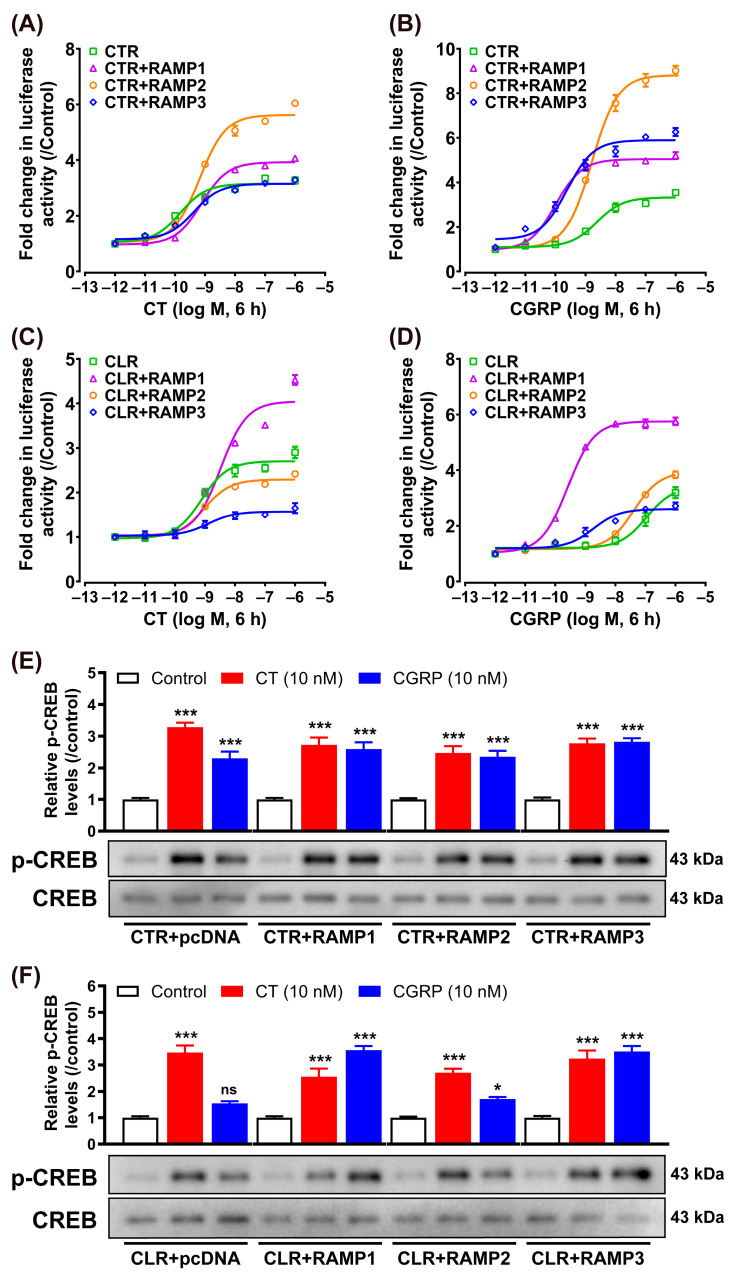
Effect of chicken CT (**A**) and CGRP (**B**) in activating chicken CTR expressed in CHO cells co-transfected with/without RAMP expression plasmid, as monitored by the pGL3-CRE-luciferase reporter system. Effect of chicken CT (**C**) and CGRP (**D**) in activating chicken CLR expressed in CHO cells co-transfected with/without RAMP expression plasmid, as monitored by the pGL3-CRE-luciferase reporter system. Data are shown as the mean ± SEM of three replicates (*n* = 3) and are representative of three independent experiments. Western blot analysis of the effect of chicken CT and CGRP treatment on the phosphorylated CREB (p-CREB) levels in CHO cells expressing chicken receptors CTR + RAMPs (**E**) and CLR + RAMPs (**F**). The cells were treated with chicken CT (10 nM, 10 min) or CGRP (10 nM, 10 min). Quantitative analysis of pCREB levels was performed using densitometry, with normalization to total CREB levels and calculation of fold change relative to the control group (0 min). Each data point represents the average of three replicates (*n* = 3) ± SEM. Representative bands of the Western blot are shown below the graph. ns, not significant; *, *p* < 0.05; ***, *p* < 0.001 vs. control (0 min).

This study initially focused on the receptor-mediated activation of the intracellular cAMP/PKA signaling pathway using the pGL3-CRE-luciferase reporter system established in our laboratory [41,42]. To determine whether the pharmacological properties of chicken CTR (or CLR) could be modified by RAMPs, we tested the responsiveness of CTR (or CLR) to their putative ligands (CT and CGRP) in the absence or presence of RAMPs (Figure 3).

In the absence of RAMPs, chicken CTR can be efficaciously activated by both ligands, and the EC_50_ values of chicken CT and CGRP in activating CTR are 0.15 ± 0.04 nM and 2.35 ± 0.63 nM, respectively. In contrast, the chicken CLR was predominantly activated by CT with an EC_50_ of 0.75 ± 0.21 nM, but it exhibited a markedly reduced sensitivity to CGRP activation, with its EC_50_ value surpassing 100 nM (Figure 3).

In the presence of RAMPs, following stimulation with chicken CT, the EC_50_ value for CTR co-expressed with RAMP1 was 0.77 ± 0.07 nM, whereas the EC_50_ values for CTR co-expressed with RAMP2 or RAMP3 were 0.67 ± 0.10 nM and 0.44 ± 0.10 nM, respectively (Figure 3A). This indicated that the presence of RAMP1, RAMP2, or RAMP3 does not markedly influence the sensitivity of chicken CTR for CT. Following stimulation with chicken CGRP, the EC_50_ value for CTR co-expressed with RAMP1 was 0.10 ± 0.01 nM, whereas the EC_50_ values for CTR co-expressed with RAMP2 or RAMP3 were 1.63 ± 0.19 nM and 0.24 ± 0.07 nM, respectively (Figure 3B). Notably, co-expression of RAMP1 or RAMP3 significantly increased the sensitivity of CTR to CGRP.

In experiments involving chicken CLR, the EC_50_ value for CLR co-expressed with RAMP1 was found to be 3.30 ± 0.96 nM following stimulation with chicken CT. For CLR co-expressed with RAMP2 and RAMP3, the EC_50_ values were 0.93 ± 0.15 nM and 1.27 ± 0.83 nM, respectively (Figure 3C). This suggests a slight reduction in chicken CLR’s sensitivity to CT in the presence of RAMP1. In the case of chicken CGRP stimulation, CLR co-expressed with RAMP1 showed an EC_50_ of 0.26 ± 0.03 nM. When chicken CLR was co-expressed with RAMP2 or RAMP3, the EC_50_ values observed were 40.86 ± 6.76 nM and 1.84 ± 0.73 nM, respectively (Figure 3D). This indicates a significant increase in CLR’s sensitivity to CGRP with RAMP1 and RAMP3 co-expression, while RAMP2 co-expression led to a limited enhancement in sensitivity.

Western blotting was also performed to confirm the above findings. It was observed that treatment with chicken CT or CGRP (10 nM, 10 min) significantly increased the CREB phosphorylation (43 kDa) of chicken CTR and CTR + RAMPs in CHO cells (Figure 3E). Furthermore, compared to peptide-free treatment (0 min), chicken CT treatment (10 nM, 10 min) significantly enhanced the CREB phosphorylation of CLR and CLR + RAMPs complexes in CHO cells, while chicken CGRP treatment (10 nM, 10 min) significantly enhanced the CREB phosphorylation of CLR + RAMP1, and CLR + RAMP3 complexes in CHO cells (Figure 3F).

### 3.4. Effect of CT/CGRP on CTR/CLR–RAMP Complexes in MAPK/ERK Signaling Pathway

**Figure 4:**
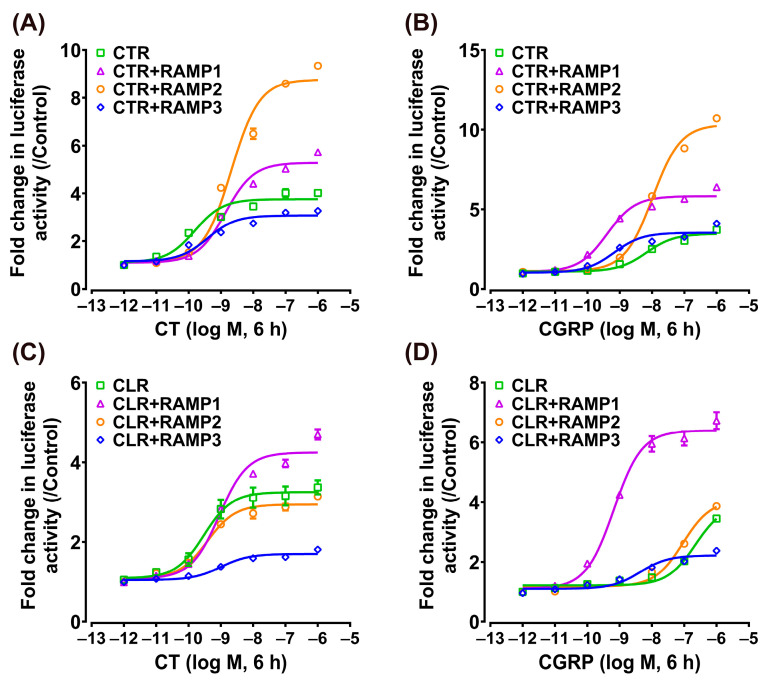
Effect of chicken CT (**A**) and CGRP (**B**) in activating chicken CTR expressed in CHO cells co-transfected with/without RAMP expression plasmid, as monitored by the pGL4-SRE-luciferase reporter system. Effect of chicken CT (**C**) and CGRP (**D**) in activating chicken CLR expressed in CHO cells co-transfected with/without RAMP expression plasmid, as monitored by the pGL4-SRE-luciferase reporter system. Data are shown as the mean ± SEM of three replicates (*n* = 3) and are representative of three independent experiments.

Using the pGL4-SRE-luciferase reporter system, we monitored the activation of the receptor-mediated intracellular MAPK/ERK signaling pathway based on our previous studies [41,43]. As shown in Figure 4, the activation dynamics observed were similar to those in the cAMP/PKA signaling pathway. Chicken CT (EC_50_: 0.15 ± 0.05 nM) and CGRP (EC_50_: 6.73 ± 1.78 nM) dose-dependently activate the individual receptor CTR, whereas the individual CLR can only be activated by CT (EC_50_: 0.30 ± 0.12 nM) and is not activated by CGRP (EC_50_ > 100 nM).

When chicken CTR was co-expressed with RAMP1, RAMP2, or RAMP3, the activation effect of CT remained potent (EC_50_: 1.38 ± 0.24 nM, 2.09 ± 0.42 nM, 0.38 ± 0.13 nM), albeit slightly weaker than that of the individual CTR (Figure 4A). Interestingly, CGRP exhibited a stronger activation effect on CTR when co-expressed with RAMP1 or RAMP3 (EC_50_: 0.41 ± 0.08 nM, 0.67 ± 0.24 nM), while its effect on CTR + RAMP2 was comparatively moderate (EC_50_: 10.38 ± 1.12 nM) (Figure 4B).

In the case of chicken CLR, when co-expressed with RAMP1, RAMP2, or RAMP3, the activation effect of CT (EC_50_: 0.89 ± 0.20 nM, 0.38 ± 0.09 nM, 1.00 ± 0.45 nM) was slightly reduced compared to its high activation effect on the individual receptor (EC_50_: 0.30 ± 0.12 nM) (Figure 4C). Conversely, co-expression with RAMPs generally tended to increase CLR’s sensitivity to CGRP. Particularly noteworthy, when chicken CLR co-existed with RAMP1 (EC_50_: 0.66 ± 0.11 nM) or RAMP3 (EC_50_: 4.31 ± 1.73 nM), CGRP’s activation potential, initially non-effective, was transformed, resulting in highly efficient receptor activation (Figure 4D).

### 3.5. Effect of CT/CGRP on CTR/CLR–RAMP Complexes in Calcium Signaling Pathway

**Figure 5:**
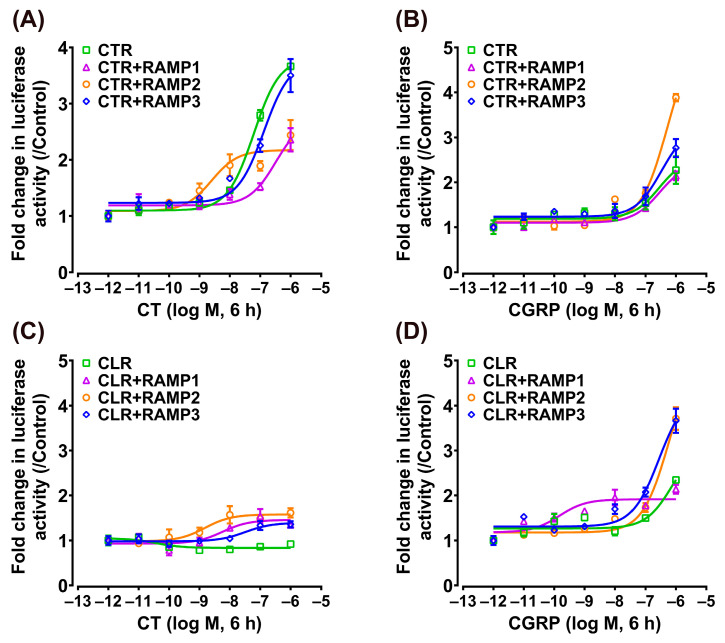
Effect of chicken CT (**A**) and CGRP (**B**) in activating chicken CTR expressed in CHO cells co-transfected with/without RAMP expression plasmid, as monitored by the pGL3-NFAT-RE-luciferase reporter system. Effect of chicken CT (**C**) and CGRP (**D**) in activating chicken CLR expressed in CHO cells co-transfected with/without RAMP expression plasmid, as monitored by the pGL3-NFAT-RE-luciferase reporter system. Data are shown as the mean ± SEM of three replicates (*n* = 3) and are representative of three independent experiments.

Utilizing the pGL3-NFAT-RE-luciferase reporter system, we also observed receptor-mediated intracellular calcium mobilization. As shown in Figure 5, chicken CGRP selectively activated CLR + RAMP1, demonstrating an EC_50_ of 0.15 ± 0.11 nM. Conversely, CT triggered activation of CTR + RAMP2 receptor and CLR + RAMP2, with EC_50_ of 2.50 ± 1.81 nM and 1.52 ± 1.32 nM, leading to a modest elevation in luciferase activity. Notably, chicken CT and CGRP exhibited negligible activation effects on other combinations of chicken CTR/CLR and RAMPs receptors, except at elevated concentrations. This pattern suggests that in CHO cells, calcium mobilization is slightly affected by the activation of chicken CTR and CLR, indicating a specific receptor–ligand interaction dynamic.

### 3.6. Tissue Distribution of CT, CGTRP, CTR, CLR, and RAMPs in Chickens

**Figure 6:**
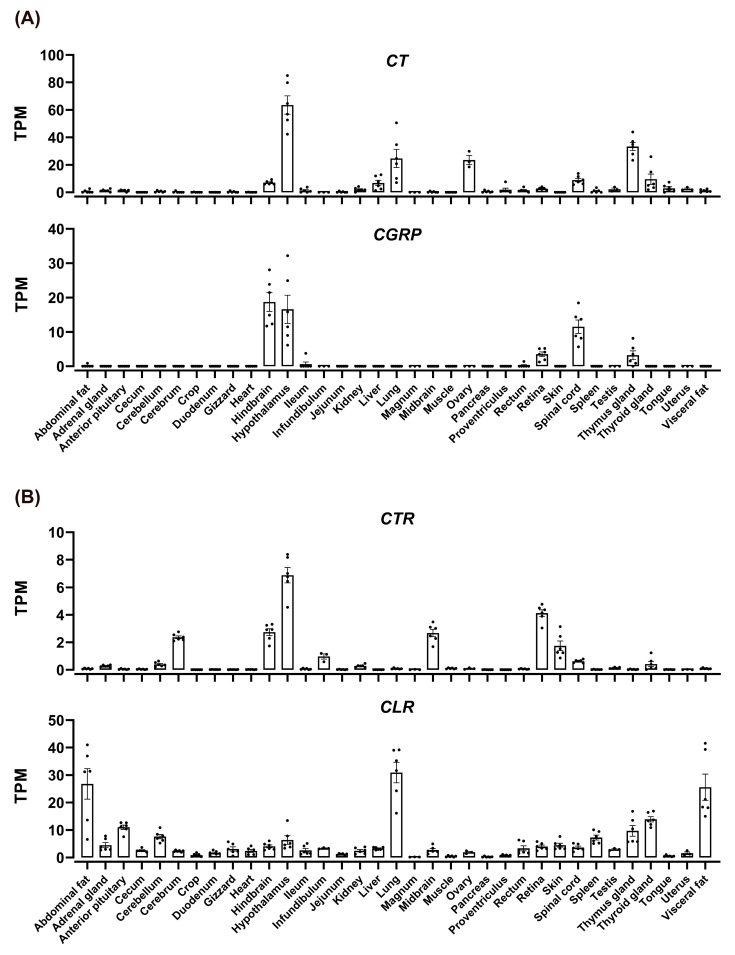
RNA-seq data analysis depicted the expression of *CT* and *CGRP* (**A**) and *CTR (CALCR)* and *CLR (CALCRL)* (**B**) in the tissues of adult Lohmann layer strain chickens. The expression levels of mRNA transcripts were estimated using transcripts per million (TPM) values. Each data point represents the mean ± SEM of six adult chickens (three males and three females; *n* = 6). However, the results for the uterus, infundibulum, magnum, and ovaries represent the mean ± SEM of three adult female chickens (*n* = 3). Similarly, the results for the testes represent the mean ± SEM of three male chickens (*n* = 3).

To examine the tissue distribution of *CT*, *CGRP*, *CTR*, *CLR*, *RAMP1*, *RAMP2*, and *RAMP3* in adult chickens, we analyzed their expression in 36 different chicken tissues using previously obtained RNA-seq data in our laboratory [40]. We found that *CT* and *CGRP* were differentially expressed in various chicken tissues (Figure 6A). *CT* was prominently expressed in the hypothalamus, lungs, thymus gland, and ovaries, with moderate expression in the hindbrain, liver, spinal cord, and thyroid gland. In contrast, *CGRP* displayed high expression in the hindbrain, hypothalamus, and spinal cord, with notable expression in the retina and thymus gland.

Investigations into the expression patterns of the receptor genes *CTR* and *CLR* disclosed distinct distribution profiles in chicken tissues (Figure 6B). *CTR* was mainly detected in the hypothalamus, retina, brain, midbrain, hindbrain, skin, infundibulum, and spinal cord. On the other hand, *CLR* was expressed in a broad range of tissues, with pronounced levels in fat, lungs, anterior pituitary, cerebellum, thyroid gland, thymus gland, and uterus. Notably, *CLR*’s expression was significantly elevated in abdominal fat and lungs compared to other tissues.

**Figure 7:**
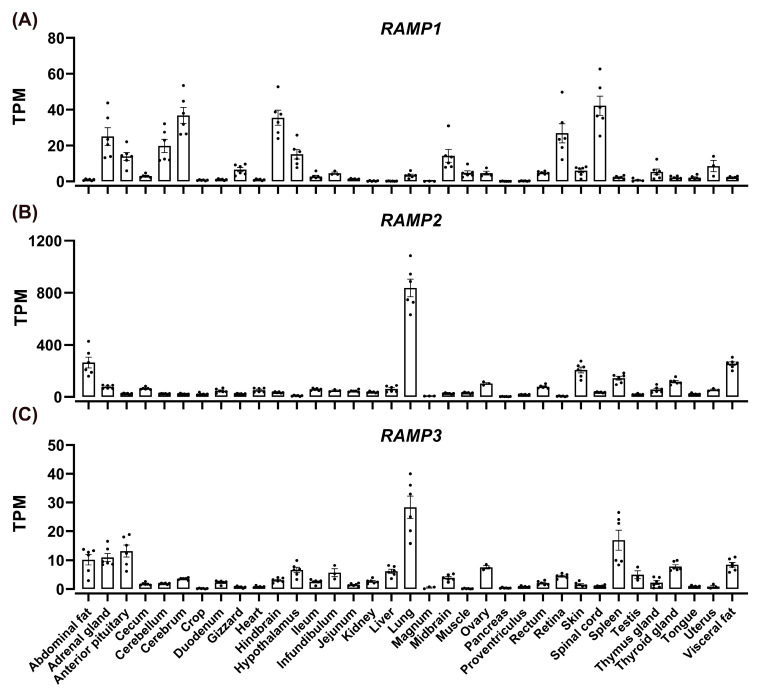
The RNA-seq data analysis depicted the expression of *RAMP1* (**A**), *RAMP2* (**B**), and *RAMP3* (**C**) in the tissues of adult Lohmann layer strain chickens. The expression levels of mRNA transcripts were estimated using TPM values. Each data point represents the mean ± SEM of six adult chickens (three males and three females; *n* = 6). However, the results for the uterus, infundibulum, magnum, and ovaries represent the mean ± SEM of three adult female chickens (*n* = 3). Similarly, the results for the testes represent the mean ± SEM of three male chickens (*n* = 3).

Furthermore, our results indicated a widespread expression of *RAMP*s in chickens (Figure 7). *RAMP1* showed high expression in the brain, hindbrain, spinal cord, cerebellum, adrenal glands, and retina. *RAMP2* was abundantly expressed in the lungs, abdominal fat, visceral fat, skin, spleen, and thyroid gland. Meanwhile, *RAMP3* was primarily found in the lungs, spleen, abdominal fat, visceral fat, adrenal glands, anterior pituitary, ovaries, and thyroid gland. Both *RAMP2* and *RAMP3* displayed considerably higher expression levels in the lungs than in other tissues.

## 4. Discussion

The calcitonin signaling systems play crucial roles in calcium homeostasis in mammals and bony fish; however, their expression and function in avian species including chicken remain poorly characterized. In this investigation, we successfully cloned the cDNAs of avian *CTR*, *CLR*, *RAMP1*, *RAMP2*, and *RAMP3*, and conducted an in-depth analysis of the functions of chicken CT and CGRP in stimulating CTR, CLR, and RAMPs in vitro. This study marks a pioneering exploration into the calcitonin signaling system within avian species, a domain previously understudied in comparison to mammals.

In this study, we cloned and identified chicken *CTR* and *CLR*. A single gene for *CALCR* and a single putative *CALCRL* gene were identified in fish, amphibians, birds, reptiles, and mammals [44]. The amino acid sequence of chicken CTR shows high homology (>90%) with avian species, but lower homology (~60%) with mammals. The homology between the cloned chicken CLR and mammalian sequences is 80–82%, higher than CTR, suggesting that the *CLR* gene is strongly conserved, while that of *CTR* is less conserved in vertebrates. The differences in the N-terminal sequences of CTR/CLR among different species may affect ligand recognition and binding, and this structure can change their affinity for ligands through interaction with RAMPs. Molecular dynamic simulations indicated that the presence of RAMPs enhances the flexibility of CTR’s extracellular N-terminal domain, which is implicated in the recognition of ligands [18].

Due to the difference in the receptor repertoire between humans and birds, the functional similarity and difference of the chicken CTR/CLR with/without RAMPs cannot be simply inferred from humans and warrants careful evaluation. Detailed studies of the pharmacology displayed by CTR and CLR, expressed with or without all three RAMPs, have been reported by several laboratories [45,46,47,48]. Because two GPCR proteins and three RAMPs can reconstitute receptors for CT and CGRP ligands, the situation is even more complex [9]. The physiological effects of CT and CGRP are mediated primarily by the ability of the receptors to couple with at least two signal transduction pathways. Coupling of CTR/CLR with the activation of the adenylate cyclase/cAMP/protein kinase A (AC/cAMP/PKA) pathway and phosphorylation of ERK1/2 has been described in several cell types in mammals [9,49]. It is also reported that Gs is a prominent route for effector coupling for CLR and CTR, but other pathways (e.g., Ca^2+^, ERK, Akt) and G proteins can be activated [50]. Therefore, three different reporter systems (pGL3-CRE-luciferase, pGL4-SRE-luciferase, and pGL3-NFAT-RE-luciferase) were used to monitor receptor-stimulated intracellular cAMP/PKA and MAPK/ERK signaling pathways and calcium mobilization in this study, respectively. In chicken, we examined all possible combinations of RAMPs and CTR/CLR, a total of eight combinations (three RAMPs and two CTR/CLR). As evidenced by luciferase reporter assays, we demonstrated that both chicken CTRs and CLRs are functional receptors, and their activation predominantly stimulates cAMP/PKA and MAPK/ERK signaling pathways, instead of calcium mobilization.

In this study, we found that chicken CTR by itself has higher sensitivity for the CT peptide, with relatively tenfold lower sensitivity interaction with CGRP. This is consistent with findings in previous studies. The CTR is a notable example of a RAMP-interacting class B GPCR that is functional in the absence of RAMPs [9,18]. Unlike CTR, co-expression with RAMPs is a prerequisite for CLR function in mammals [13,49]. Interestingly, our findings demonstrated that chicken CLR by itself can serve as a functional receptor for CT in nanomolar concentrations (~0.75 nM), instead of CGRP (>100 nM). It is well known that CLR by itself binds no known endogenous ligand, but in the presence of RAMPs, it gives receptors for CGRP in mammals [17,51]. Our results indicated that there may be species differences in receptor pharmacology for chicken CLR. The mechanisms behind these differences need further research. Recently, structures of the CGRP receptor (CLR + RAMP1) ECDs bound to a CGRP analogue were determined [52]. However, as the co-expression of RAMPs is essential for the expression and proper function of CLR, it provides only limited insight into how RAMPs modify CLR to allow novel ligand pharmacology [18]. The chicken CLR is functional in the absence of RAMPs, which may make it an ideal system to probe how RAMP interaction enables CLR function or the role of CLR ECD residues in peptide–ligand interactions in the absence and presence of each RAMP.

The function and pharmacology of CTR and CLR are altered in the presence of RAMPs, which are single-TM-domain proteins, identified as a family of three members: RAMP1, RAMP2, and RAMP3 [13]. Remarkably, our results indicated that the presence of RAMP1, RAMP2, or RAMP3 resulted in a slight decrease in sensitivity towards CT, with a minimal effect in activating adenylate cyclase. This differs from the results observed in mammals, where mammalian CTR does not exhibit a significant response to CT stimulation in the presence of RAMPs [13,50,53]. For the CGRP peptide, the sensitivity of CTR is ranked in the following order: CTR + RAMP1 ≈ CTR + RAMP3 > CTR + RAMP2 ≈ CTR. This highlights the role of RAMP1 and RAMP3 in markedly increasing the sensitivity of CTR to CGRP, while RAMP2 has a lesser impact. The results demonstrate that the sensitivity of chicken CTR to CT and CGRP is influenced by the type of RAMP it is associated with. The response to CT is primarily determined by CTR itself, whereas a heightened sensitivity to CGRP requires the presence of either RAMP1 or RAMP3. These findings reveal the critical role of chicken RAMPs in modulating the response of CTR to different peptide hormones, especially in enhancing the response to CGRP.

In mammals, CTR functions as a receptor for three distinct hormones: CT, CGRP, and amylin (not explored in this study). The specific affinity spectrum is determined by its co-expression with RAMPs. Although CTR alone is a receptor for CT that is involved in bone homeostasis, CTRs in complex with RAMP1–3 are high-affinity receptors for amylin, a peptide involved in the regulation of food intake [54]. RAMP1- and RAMP3-derived amylin receptors demonstrate the highest affinity for salmon CT, moderate affinity for rat amylin, and low affinity for human CT [51,53]. The CTR + RAMP1 complex is also a high-affinity receptor for the neuropeptide CGRP. However, RAMP3-derived CTR receptor complexes had significantly lower affinity for CGRP than those derived from RAMP1 [55,56]. Interestingly, our study proposed the presence of a novel chicken CGRP-responsive receptor (CTR + RAMP3), which exhibits a sensitivity to CGRP that is comparable to the sensitivity observed in the traditionally recognized “classical CGRP receptor”, the CTR + RAMP1 complex. A further understanding of the role of the CTR + RAMP3 complex in CGRP biology in chicken is urgently required.

For CLR, the sensitivity to CT is ranked in the following order: CLR ≈ CLR + RAMP2 ≈ CLR + RAMP3 > CLR + RAMP1 (EC_50_: 0.75–3.30 nM). This showed that RAMP2 and RAMP3 did not affect sensitivity, whereas RAMP1 slightly reduced sensitivity to CT. In contrast to mammal CLR, which is dependent on RAMPs for cell surface expression, chicken CLR may transport to the cell surface on its own and function independently/dependently of RAMPs as the receptor for CT. Further experimental studies are required to substantiate and investigate these findings. On the contrary, CLR alone does not show a significant response to CGRP stimulation (>100 nM) at physiological concentrations (~1 nM). The dimerization of CLR with RAMP1 shows the highest sensitivity for CGRP and corresponds to the previously characterized CTR + RAMP1 and CTR + RAMP3 complexes in pharmacological activity (~0.2 nM). The sensitivity to CGRP is ranked in the following order: CLR + RAMP1 > CLR + RAMP3 >> CLR + RAMP2 >> CLR. This indicated that only in the presence of RAMP1 and RAMP3 could CLR + RAMP complexes act as functional receptors for CGRP. In the presence of RAMP1, the EC_50_ values of CGRP were approximately tenfold lower than CT, which is consistent with previous studies on humans. It has been demonstrated that RAMP1 is required to transport CLR to the cell surface in order to form a functional receptor able to become bound and activated by CGRP [17].

**Figure 8:**
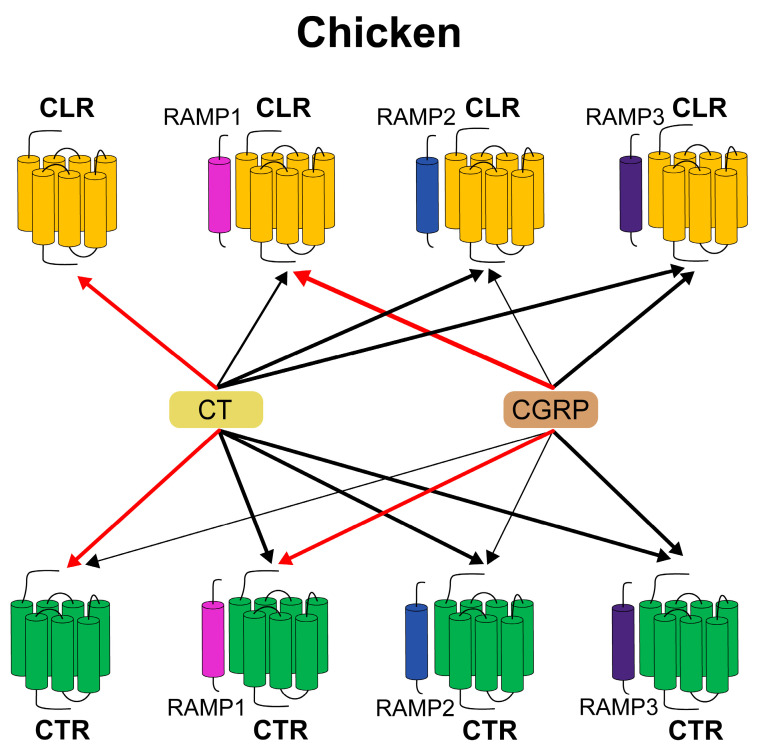
The interaction patterns between ligands (CT, CGRP) and receptors (CTR, CLR) influenced by RAMPs for cAMP production in chickens. Specifically, chicken CTR and CLR can associate with RAMPs to form eight distinct functional receptors. Chicken CT strongly activates independent CTR and CLR, whereas CGRP preferentially activates CTR with no effect on CLR. When combined with RAMPs, CTR and CLR can be simultaneously activated by CT and CGRP, with the presence of RAMP1 and RAMP3 significantly enhancing CGRP’s activation of CTR and CLR. Chicken CT shows the capability to stimulate all eight receptor configurations, including the standalone receptors as well as those in complex with various RAMPs. The red lines in the figure indicate receptors corresponding to CT and CGRP with the highest activation potential.

Our data suggest a significant divergence in the functional characteristics of chicken CTR and CLR when compared with their mammalian counterparts. Currently, receptor function investigations have been primarily conducted on non-mammalian species, notably *Xenopus tropicalis* [57] and *Takifugu obscurus* [58,59], representing a limited scope of information to fully explain the mechanism of species-specific differences. Here, we provide a preliminary insight into the intricate relationship between CLR, CT, CGRP, and RAMPs (Figure 8). Further research is necessary for a more comprehensive understanding of their interactions.

In this study, we also identified the differential expression of *CT*, *CGRP*, *CTR*, *CLR*, and *RAMPs* in various tissues of adult chickens, highlighting their distinct roles in avian biology. Our findings were consistent with previous studies that reported the existence of *CT* in the bird’s hypothalamus [60,61], and CGRP-ir was widely distributed in neurons or neuronal fibers of the CNS [30]. There are also reports indicating that central injection of CT or CGRP can induce satiety [32,62]. In humans, *CT* is also predominantly found in the lungs [63,64]. Similarly, chicken *CT* is highly expressed in the lungs and may exert paracrine effects within the lung tissue by modulating intracellular and transcellular calcium movements [65]. Interestingly, in chickens, the expression of *CT* in the ovaries hints at a potentially more prominent role in reproduction compared to mammals, as it is expressed in granulosa cells and follicle membrane cells, potentially regulating follicular maturation [61]. CT may inhibit progesterone synthesis stimulated by luteinizing hormone in the largest granulosa cells of the hen’s follicles, thereby regulating eggshell formation [66].

*CTR* and *CLR* are widely expressed in chickens, with the former implicated in hypothalamic appetite regulation similar to mammals and subject to ovulation-related changes in the hen hypothalamus [32,62,67]. CT also appears to influence adrenocorticotropic hormone (ACTH) secretion, related to corticotropin-releasing hormone (CRH) activity in the anterior pituitary [68], where CLR’s high expression may indicate a role in hormone regulation. In human and rat studies, CLR is linked to vascular and inflammatory responses [69,70,71], and given its lung expression in chickens, it might share these roles. Furthermore, CLR’s presence in chicken adipose tissue aligns with findings linking it to lipid metabolism and obesity management, suggesting it as a gene of interest for fat deposition [72,73,74].

Utilizing transcriptome sequencing, we quantitatively compared the expression levels of the three *RAMP* genes. We revealed that *RAMP2* exhibited the highest expression levels in many tissues, denoted by a transcripts per million (TPM) value of over 200. *RAMP1* transcripts predominate in the nervous system and adrenal gland, enabling the regulation of CLR’s affinity for CGRP. CGRP displays robust activity in cerebral circulation, potentially modulating chicken neuronal activity [75] and inducing vasodilation [76]. *RAMP2* and *RAMP3* transcripts predominate in the lungs, where *RAMP1* expression is low, indicating their interaction with CLR in the lungs, receiving stimulation from CT and CGRP, and participating in calcium regulation and stress protection in the cardiovascular system [9]. Binding of CLR in adipose tissues has been linked to the regulation of fat metabolism [51,77]. Considering the notable expression of *CLR*, *RAMP2*, and *RAMP3* in chicken adipose tissue, further investigations are necessary to elucidate their detailed roles in chicken fat metabolism.

## 5. Conclusions

In summary, our study has shed light on the intricate calcitonin signaling system within chickens, an area that has been little explored in avian species. We cloned and analyzed the cDNAs of *CTR*, *CLR*, *RAMP1*, *RAMP2*, and *RAMP3* in chickens. In vitro analyses demonstrated that the cAMP/PKA and MAPK/ERK pathways are the primary downstream signaling pathways for chicken CTR and CLR activation. Remarkably, chicken CLR can act as a functional receptor for CT in the absence/presence of RAMPs. The study uncovers evolutionary divergences in receptor pharmacology, including the unique functionality of chicken CLR for CT and differing sensitivities of CTR influenced by RAMPs. Additionally, we observed broad and differential tissue-specific expression of *CT*, *CGRP*, *CTR*, *CLR*, and *RAMP*s, hinting at diverse physiological impacts across chicken tissues. This work enhances our understanding of these systems in chickens compared to mammals and lays a foundational perspective for further investigations into their roles in vertebrate biology.

## Figures and Tables

**Table 1:** EC_50_ values of chicken CT and CGRP in activating the cAMP/PKA, MAPK/ERK, and calcium signaling pathways in CHO cells expressing different receptors.

EC_50_ Values (nM)					
cAMP/PKA signaling pathway					
	CT	CGRP		CT	CGRP
CTR + pcDNA	0.15 ± 0.04	2.35 ± 0.63	CLR + pcDNA	0.75 ± 0.21	—
CTR + RAMP1	0.77 ± 0.07	0.10 ± 0.01	CLR + RAMP1	3.30 ± 0.96	0.26 ± 0.03
CTR + RAMP2	0.67 ± 0.10	1.63 ± 0.19	CLR + RAMP2	0.93 ± 0.15	40.86 ± 6.76
CTR + RAMP3	0.44 ± 0.10	0.24 ± 0.07	CLR + RAMP3	1.27 ± 0.83	1.84 ± 0.73
MAPK/ERK signaling pathway					
	CT	CGRP		CT	CGRP
CTR + pcDNA	0.15 ± 0.05	6.73 ± 1.78	CLR + pcDNA	0.30 ± 0.12	—
CTR + RAMP1	1.38 ± 0.24	0.41 ± 0.08	CLR + RAMP1	0.89 ± 0.20	0.66 ± 0.11
CTR + RAMP2	2.09 ± 0.42	10.38 ± 1.12	CLR + RAMP2	0.38 ± 0.09	90.93 ± 17.36
CTR + RAMP3	0.38 ± 0.13	0.67 ± 0.24	CLR + RAMP3	1.00 ± 0.45	4.31 ± 1.73
Calcium signaling pathway					
	CT	CGRP		CT	CGRP
CTR + pcDNA	61.10 ± 8.55	—	CLR + pcDNA	NA ^a^	—
CTR + RAMP1	—	—	CLR + RAMP1	5.81 ± 5.75	0.15 ± 0.11
CTR + RAMP2	2.50 ± 1.81	—	CLR + RAMP2	1.52 ± 1.32	—
CTR + RAMP3	—	—	CLR + RAMP3	32.3 ± 30.3	—

Em dashes denote an EC_50_ value > 100 nM based on the experimental data. ^a^ NA indicates that the EC_50_ value could not be estimated based on the experiment data.

## Data Availability

The supporting data of this study are available within the article.

## Supplementary Materials

The following supporting information can be downloaded at https://www.mdpi.com/article/10.3390/ani14071058/s1. Table S1: Primers used in this study.

## Abbreviations

AC—adenylyl cyclase; ACTH—adrenocorticotropic hormone; Akt—protein kinase B; AM—adrenomedullin; AMY—amylin; CGRP—calcitonin gene-related peptide; CLR—calcitonin receptor-like receptor; CNGB—China National GeneBank; CNSA—China National GeneBank Sequence Archive; CRE—cAMP response element; CREB—cAMP response element-binding protein; CRH—corticotropin-releasing hormone; CTR—calcitonin receptor; DEPC—diethyl pyrocarbonate; dNTP—deoxyribonucleoside triphosphate; ECD—extracellular domain; GPCRs—G protein-coupled receptors; mAb—monoclonal antibody; MAPK—mitogen-activated protein kinase; MMLV—Moloney murine leukemia virus; NFAT-RE—nuclear factor of activated T cells—response element; NGBdb—National GeneBank DataBase; ORF—open reading frame; p-CREB—phosphorylated CREB; PKA—protein kinase A; RAMPs—receptor activity-modifying proteins; RNA-seq—RNA sequencing; SRE—serum response element; TGEA—tissue gene expression atlas; TM—transmembrane domain; TPM—transcripts per million.
