# Supplementary material for: Functional Analysis and Tissue-Specific Expression of Calcitonin and CGRP with RAMP-Modulated Receptors CTR and CLR in Chickens
Source: Animals (Basel). 2024 Mar 30;14(7):1058. doi: 10.3390/ani14071058 (PMC11010885; doi:10.3390/ani14071058)
Supplement: Supplementary file 1 [file animals-14-01058-s001.zip › animals-2905275-supplementary.pdf]

Supplemental Table S1: Primers used in this study.

Gene	Sense/antisense	Primer sequence (5'-3')
CTR	Sense	TGGTGGAATTCGCCACCATGAAAAAAACAACCCACAG
	Antisense	GCTGGATATCTGCAGAATTCCTAGGCTGATGTCTCCCCA
CLR	Sense	TGGTGGAATTCGCCACCATGACAAAAAACTGGATCAC
	Antisense	GCTGGATATCTGCAGAATTCCTACACTGTCATGTCAT
RAMP1	Sense	TTGGTACCGAGCTCGGATCCGCCACCATGGCCCTGCTGCCGCGCCGCTTC
	Antisense	TGCTGGATATCTGCAGAATTCCTCACTGCCACCAGCATCCCCAGGT
RAMP2	Sense	TTGGTACCGAGCTCGGATCCGCCACCATGGCACCGTGTGCGCAGATGG
	Antisense	GCTGGATATCTGCAGAATTCCTAAGGCTGTGCCTTGCCGT
RAMP3	Sense	TGGTGGAATTCGCCACCATGGAGGCGTCCGGCCGCTGCC
	Antisense	GTGGATCCATTGGGAACAACTTACCCTAGAATATCACTTC

All primers were synthesized by Youkang Biotechnology (Chengdu, China)
